# Multi-Model Minimum Error Entropy Recursive Three-Step Filter

**DOI:** 10.3390/e28070750

**Published:** 2026-07-01

**Authors:** Xiaoliang Feng, Jiawei Zhang

**Affiliations:** School of Electrical and Energy Engineering, Shanghai Dianji University, Shanghai 201306, China

**Keywords:** recursive three-step filter, minimum error entropy, unknown input estimation, multi-model filtering, nonlinear systems, non-Gaussian noise

## Abstract

This paper investigates state estimation for strongly nonlinear systems with unknown inputs under non-Gaussian heavy-tailed impulsive noise. Conventional recursive three-step filters (RTSF) based on the minimum-variance criterion are sensitive to outliers, while a single local linearization is often inadequate for strongly nonlinear dynamics. To overcome these limitations, a multi-model minimum error entropy recursive three-step filter (MMMEERTSF) is proposed. The minimum error entropy criterion is embedded into the RTSF framework to enhance robustness against abnormal disturbances, and iterative reweighted solutions are developed for unknown-input estimation and state correction by combining residual whitening with entropy-based optimization. Meanwhile, multiple local linear submodels are constructed to approximate the nonlinear system, and compatibility-based posterior fusion is employed to obtain the final estimate. The proposed method shows improved robustness and competitive estimation accuracy under non-Gaussian mixture and impulsive noise, especially in the nonlinear multi-model case.

## 1. Introduction

State estimation for systems with unknown inputs arises in many engineering applications, such as motor drives, spacecraft attitude control, navigation and positioning, and industrial process monitoring [1,2,3]. For such systems, the recursive three-step filter (RTSF) provides a clear and structured estimation framework by decomposing the recursive procedure into unknown-input estimation, state correction, and covariance propagation [2,3,4,5,6]. However, most existing RTSF methods are developed under the minimum-variance criterion and inherently rely on second-order error statistics. When the system is affected by heavy-tailed noise, impulsive disturbances, or outliers, the conventional quadratic cost can be dominated by abnormal samples, resulting in increased estimation bias and even filter degradation or divergence [7,8,9,10,11].

On the other hand, practical systems usually exhibit significant nonlinearities in both the state and measurement equations. For such systems, methods based on a single local linearization, such as the EKF and its RTSF-type extensions, may fail to provide sufficient approximation accuracy when the operating point changes rapidly or the system evolves in strongly nonlinear regions, thereby limiting the filtering performance [12,13,14,15,16]. To improve estimation accuracy for nonlinear systems, multi-model and multi-linearization strategies have attracted increasing attention. Existing studies have shown that fusion-based estimators constructed from multiple local models can describe nonlinear dynamics more effectively and improve estimation performance [13,17]. Therefore, in the presence of unknown inputs, it remains challenging to simultaneously suppress non-Gaussian abnormal disturbances and reduce modeling errors caused by strong nonlinearities. More specifically, this problem involves three coupled difficulties. First, the direct feedthrough of unknown inputs links unknown-input estimation with state correction, causing errors to propagate between the two steps. Second, heavy-tailed, impulsive, or mixed non-Gaussian measurement noises may generate abnormal residuals and degrade the robustness of the quadratic-cost-based RTSF. Third, for strongly nonlinear systems, a single local linearization may lead to noticeable approximation errors when the operating point changes rapidly. Therefore, a robust RTSF framework is needed to handle unknown-input coupling, non-Gaussian disturbances, and nonlinear approximation errors simultaneously.

To enhance robustness under non-Gaussian noise, several robust criteria, such as the Huber criterion, the maximum correntropy criterion (MCC), and the minimum error entropy (MEE) criterion, have been widely studied [7,8,9,10,18,19,20,21,22]. Among them, the Huber criterion reduces the influence of large residuals through a piecewise penalty mechanism, while MCC improves robustness against outliers and impulsive noise by measuring local similarity in a kernel space. In contrast, MEE characterizes the distribution of estimation errors from the perspective of information-theoretic learning and is more suitable for heavy-tailed, impulsive, and mixed non-Gaussian noise environments than criteria relying only on second-order moments [10,19,20,21,22]. Recently, MEE- and MCC-based robust filters have also been further extended to graph-based filtering, high-order nonlinear filtering, continuous-discrete filtering, distributed estimation, integrated navigation, and nonlinear state estimation under complex non-Gaussian noise [23,24,25,26,27,28,29]. In addition, robust adaptive filtering studies in active noise control have also shown that conventional quadratic-error-based algorithms may suffer from performance degradation under impulsive noise, whereas robust cost functions such as MCC can improve the stability and robustness of adaptive filters [30]. Although entropy-based criteria have been introduced into KF, EKF, UKF, and CKF filtering frameworks, studies on MEE-based robust estimation within the recursive three-step structure remain limited, especially for systems with unknown inputs and strong nonlinearities. In particular, a unified multi-model extension for RTSF under the MEE criterion is still lacking.

To address the above issues, this paper proposes a multi-model minimum error entropy recursive three-step filter (MMMEERTSF) for state estimation of strongly nonlinear systems with unknown inputs under non-Gaussian heavy-tailed impulsive noise. The proposed method incorporates the MEE criterion into the RTSF framework and constructs an error-entropy-based objective function using whitened residuals, such that both unknown-input estimation and state correction can be uniformly formulated as iterative reweighted solutions to enhance the suppression of abnormal residuals. Meanwhile, multiple local linear submodels are established around the current estimate, and the final estimate is obtained through compatibility-based posterior fusion, thereby improving the approximation capability for strongly nonlinear dynamics. Simulation results demonstrate that the proposed method outperforms several representative RTSF-family filters in terms of estimation accuracy, robustness, and convergence performance.

## 2. Background

The following nonlinear system with unknown input is considered:

(1) $$x_{k}=f\left(x_{k-1},d_{k-1}\right)+q_{k-1}$$

(2) $$y_{k-1}=h\left(x_{k-1},d_{k-1}\right)+r_{k-1}$$

where $x_{k}\in R^{n}$, $d_{k-1}\in R^{m}$, and $y_{k-1}\in R^{p}$ are the state vector, the unknown input vector, and the measurement vector at time k−1, respectively. f(⋅) and h(⋅) are known nonlinear functions. The process noise $q_{k-1}$ and the measurement noise $r_{k-1}$ are assumed to be zero-mean random sequences with covariance matrices $Q_{k-1}$ and $R_{k-1}$, respectively. In addition, $q_{k-1}$, $r_{k-1}$, and the initial state $x_{0}$ are assumed to be mutually independent. This paper does not impose any prior statistical assumption on the unknown input $d_{k-1}$, and its type or variation law is not specified in advance.

Based on this system, this paper aims to address the state estimation problem for nonlinear systems with unknown inputs under heavy-tailed impulsive noise. To this end, a recursive three-step filtering method based on the minimum error entropy criterion is developed, and fixed-point iteration is employed to realize the joint estimation of the unknown input and the system state.

For nonlinear systems, the state and measurement equations are nonlinear, which prevents the recursive three-step filtering framework from being directly applied. Therefore, following the conventional idea of the extended RTSF, the nonlinear functions are approximated by first-order Taylor expansion around prescribed local reference points at each time instant.

In the single-point RTSF, only one local linearization point is used, i.e., i=1. In the multi-model extension, several local expansion points are introduced, and each value of i corresponds to one local linearized model. The estimates obtained from these local models are then fused to improve the robustness against linearization errors.

It should be emphasized that the reference value $d_{k-1}$ is only used as the local expansion point for the unknown input. It may be selected from the previous estimate or from the initial value of the fixed-point iteration, and it does not imply any prior statistical distribution of the unknown input.

(3) $$\begin{array}{c} x_{k}\approx f\left({\hat{x}}_{k-1|k-2},{\hat{d}}_{k-1|k-2}\right)+A_{k-1}\left(x_{k-1}-{\hat{x}}_{k-1|k-2}\right)\\ +B_{k-1}\left(d_{k-1}-{\hat{d}}_{k-1|k-2}\right)+q_{k-1}\; \end{array}$$

(4) $$\begin{array}{c} \;y_{k-1}\approx h\left({\hat{x}}_{k-1|k-2},{\hat{d}}_{k-1|k-2}\right)+C_{k-1}\left(x_{k-1}-{\hat{x}}_{k-1|k-2}\right)\\ +D_{k-1}\left(d_{k-1}-{\hat{d}}_{k-1|k-2}\right)+r_{k-1} \end{array}$$

where

$$A_{k-1}=\frac{\partial f\left(x,d\right)}{\partial x}{|}_{\left({\hat{x}}_{k-1|k-2},{\hat{d}}_{k-1|k-2}\right)}\;B_{k-1}=\frac{\partial f(x,d)}{\partial d_{k-1}}{|}_{\left({\hat{x}}_{k-1|k-2},{\hat{d}}_{k-1|k-2}\right)}$$

$$C_{k-1}^{i}=\frac{\partial h(x,d)}{\partial x}{|}_{\left({\hat{x}}_{k-1|k-2},{\hat{d}}_{k-1|k-2}\right)}\;D_{k-1}^{i}=\frac{\partial h(x,d)}{\partial d_{k-1}}{|}_{\left({\hat{x}}_{k-1|k-2},{\hat{d}}_{k-1|k-2}\right)}$$

By rearranging the above expressions, the system can be rewritten as

(5) $$x_{k}=A_{k-1}x_{k-1}+B_{k-1}d_{k-1}+\xi_{k-1}+q_{k-1}$$

(6) $$y_{k-1}=C_{k-1}x_{k-1}+D_{k-1}d_{k-1}+\varsigma_{k-1}+r_{k-1}$$

where

$$\xi_{k-1}=f\left({\hat{x}}_{k-1|k-2},{\hat{d}}_{k-1|k-2}\right)-A_{k-1}{\hat{x}}_{k-1|k-2}-B_{k-1}{\hat{d}}_{k-1|k-2}$$

$$\varsigma_{k-1}=h\left({\hat{x}}_{k-1|k-2},{\hat{d}}_{k-1|k-2}\right)-C_{k-1}{\hat{x}}_{k-1|k-2}-D_{k-1}{\hat{d}}_{k-1|k-2}$$

$\xi_{k-1}$ and $\varsigma_{k-1}$ are deterministic affine compensation terms generated by local linearization, rather than stochastic noise components. In each local branch, the Jacobian matrices and affine compensation terms are fixed at the selected expansion point during the current recursion.

### 2.1. Recursive Three-Step Filter

For the locally linearized affine model with direct feedthrough of the unknown input in the measurement equation, the recursive three-step filter (RTSF) is given as follows. Since $\varsigma_{k-1}^{i}$ is a known affine term contained in the predicted measurement, it should be subtracted when constructing the measurement residual. Otherwise, this deterministic linearization bias would enter the unknown-input estimation and state correction. It is assumed that $D_{k-1}^{i}$ has full column rank.

Step 1: Unknown Input Estimation.

(7) $${\tilde{R}}_{k-1}=C_{k-1}P_{k-1|k-2}^{x}C_{k-1}^{T}+R_{k-1}$$

(8) $$M_{k-1}={\left(D_{k-1}^{T}{\tilde{R}}_{k-1}^{-1}D_{k-1}\right)}^{-1}D_{k-1}^{T}{\left({\tilde{R}}_{k-1}\right)}^{-1}$$

(9) $${\hat{d}}_{k-1}=M_{k-1}\left(y_{k-1}-\varsigma_{k-1}-C_{k-1}{\hat{x}}_{k-1|k-2}\right)$$

(10) $$P_{k-1}^{d}={\left(D_{k-1}^{T}{\tilde{R}}_{k-1}^{-1}D_{k-1}\right)}^{-1}$$

Step 2: Measurement Update

(11) $$K_{k-1}=P_{k-1|k-2}^{x}C_{k-1}^{T}{(\tilde{R}}_{k-1}{)}^{-1}$$

(12) $${\hat{x}}_{k-1|k-1}={\hat{x}}_{k-1|k-2}+K_{k-1}\left(y_{k-1}-\varsigma_{k-1}-C_{k-1}{\hat{x}}_{k-1|k-2}-D_{k-1}{\hat{d}}_{k-1}\right)$$

(13) $$P_{k-1|k-1}^{x}=P_{k-1|k-2}^{x}-K_{k-1}\left({\tilde{R}}_{k-1}-D_{k-1}P_{k-1}^{d}D_{k-1}^{T}\right)K_{k-1}^{T}$$

(14) $$P_{k-1}^{xd}={\left(P_{k-1}^{dx}\right)}^{T}=-K_{k-1}D_{k-1}P_{k-1}^{d}$$

Step 3: Time Update

(15) $${\hat{x}}_{k|k-1}=A_{k-1}{\hat{x}}_{k-1|k-1}+B_{k-1}{\hat{d}}_{k-1}+\xi_{k-1}$$

(16) $$P_{k|k-1}^{x}=\left[\begin{array}{cc} A_{k-1} & B_{k-1} \end{array}\right]\left[\begin{array}{cc} P_{k-1|k-1}^{x} & P_{k-1}^{xd}\\ P_{k-1}^{dx} & P_{k-1}^{d} \end{array}\right]\left[\begin{array}{c} A_{k-1}^{T}\\ B_{k-1}^{T} \end{array}\right]+Q_{k-1}$$

The above three-step recursion provides a structured framework for simultaneous unknown-input and state estimation. No prior probability distribution or dynamic model is assumed for the unknown input. The quantities ${\bar{d}}_{k-1}$ and $P_{k-1}^{d}$ are introduced only for fixed-point initialization, residual whitening, and weighting of the unknown-input residual, rather than representing statistical prior information about the true unknown input.

### 2.2. Minimum Error Entropy of Gaussian Kernel

Minimum error entropy (MEE) criterion: let the error between two random variables X and Y be defined as e=X−Y. In the MEE framework, the uncertainty of the error can be measured by Rényi’s entropy, i.e.,

(17) $$H_{\alpha }\left(e\right)=\frac{1}{1-\alpha }logV_{\alpha }\left(e\right)$$

where α is the order of Rényi’s entropy, and $V_{\alpha }\left(e\right)$ represents the information potential, which is defined as

(18) $$V_{\alpha }\left(e\right)=\int P^{\alpha }\left(e\right)de=E\left[P^{\alpha -1}\left(e\right)\right]$$

where p(.) is the probability density function (PDF) of the error ***e***, and E(.) represents the expectation operator. In practical applications, the error PDF is usually unknown and can be estimated by the Parzen window method as

(19) $$\hat{p}\left(e\right)=\frac{1}{N}\sum_{j=1}^{N}G_{\sigma }\left(e-e_{j}\right)$$

where $G_{\sigma }$ is the Gaussian kernel function, defined as,

(20) $$G_{\sigma }\left(u\right)={\frac{1}{\sqrt{2\pi }\sigma }e}^{-\frac{u^{2}}{2\sigma^{2}}}$$

where σ is the kernel width.

In this paper, we take α=2, from which the sample estimate of the second-order information potential can be obtained as

(21) $$\hat{V_{2}}\left(e\right)=\frac{1}{N}\sum_{i=1}^{N}\hat{p}\left(e_{i}\right)=\frac{1}{N^{2}}\sum_{i=1}^{N}\sum_{j=1}^{N}G_{\sigma }\left(e_{i}-e_{j}\right)$$

For the Gaussian kernel, the kernel value decreases exponentially as the pairwise error difference increases. Therefore, error samples with large deviations contribute much less to the information potential, which enables the MEE criterion to suppress the influence of outliers and impulsive disturbances. This property makes MEE suitable for robust estimation under non-Gaussian noise.

**Remark 1.**  *Practical verification of the assumptions.*

In practical applications, the above assumptions can be checked or approximately verified from the system model and measured data. The dimensional and rank conditions can be examined by matrix-rank calculation. The positive definiteness of covariance matrices can be checked by Cholesky decomposition. The independence of process and measurement noises can be approximately assessed by residual correlation analysis. For nonlinear systems, the validity of local linearization can be evaluated from the state variation and linearization residual within one sampling interval. When these assumptions are only approximately satisfied, the MEE criterion and the multi-model structure can improve the robustness of the filter.

## 3. Derivation of the MEERTSF Algorithm

### 3.1. Unknown Input Estimation Based on MEE

Define the generalized observation residual after linearization as ${\tilde{y}}_{k-1}^{*}=y_{k-1}-C_{k-1}{\hat{x}}_{k-1|k-2}^{*}$, where ${\hat{x}}_{k-1|k-2}^{*}$ denotes the one-step state prediction obtained from the previous step, and $C_{k-1}=\frac{\partial h(x_{k-1},d_{k-1})}{\partial x_{k-1}}{|}_{\left({\hat{x}}_{k-1|k-2}^{i},{\hat{d}}_{k-1|k-2}^{i}\right)}$, and its covariance matrix is denoted by $P_{k-1|k-2}^{x*}$, which represents the one-step state prediction error covariance matrix under the MEE criterion. Then, ${\tilde{x}}_{k-1|k-2}^{*}=x_{k-1}-{\hat{x}}_{k-1|k-2}^{*},$ and by substituting it into the observation equation of the system model in (6), one obtains

(22) $$y_{k-1}=C_{k-1}\left({\tilde{x}}_{k-1|k-2}^{*}+{\hat{x}}_{k-1|k-2}^{*}\right)+D_{k-1}d_{k-1}+{\bar{r}}_{k-1}$$

Rearranging (22), the following pseudo-measurement equation with respect to the unknown input can be obtained:

(23) $$\left[\begin{array}{c} {\bar{d}}_{k-1}\\ y_{k-1}-C_{k-1}{\hat{x}}_{k-1|k-2}^{*}-\varsigma_{k-1} \end{array}\right]=\left[\begin{array}{c} I_{m}\\ D_{k-1} \end{array}\right]d_{k-1}+\left[\begin{array}{c} -\left(d_{k-1}-{\bar{d}}_{k-1}\right)\\ C_{k-1}{\tilde{x}}_{k-1|k-2}^{*}+r_{k-1} \end{array}\right]$$

To express (23) in a compact regression form, the terms independent of the unknown input estimate are collected into an augmented noise vector, which is defined as

(24) $$\left[\begin{array}{c} -\left(d_{k-1}-{\bar{d}}_{k-1}\right)\\ C_{k-1}{\tilde{x}}_{k-1|k-2}^{*}+r_{k-1} \end{array}\right]=v_{k-1}^{*}$$

where $d_{k-1}$ denotes the unknown input to be estimated, ${\bar{d}}_{k-1}$ is its prior value or initial guess, ${\tilde{x}}_{k-1|k-2}^{*}=x_{k-1}-{\hat{x}}_{k-1|k-2}^{*}$ denotes the local state prediction error, $r_{k-1}$ is the measurement noise, and $v_{k-1}^{*}$ is the augmented noise vector containing the unknown-input linearization error, the state prediction error, and the measurement noise. According to (24), the covariance matrix of the augmented noise vector is given by

(25) $$E\left[v_{k-1}^{*}v_{k-1}^{*T}\right]=\left[\begin{array}{cc} P_{k-1}^{d} & 0\\ 0 & {\tilde{\hat{R}}}_{k-1} \end{array}\right]=\left[\begin{array}{cc} G_{k-1}^{d}G_{k-1}^{dT} & 0\\ 0 & {\tilde{G}}_{k-1}^{r}{\tilde{G}}_{k-1}^{rT} \end{array}\right]=G_{k-1}^{*}G_{k-1}^{*T}$$

Note that $G_{k-1}^{d}$ and ${\tilde{G}}_{k-1}^{r}$ are obtained from $P_{k-1}^{d}$ and ${\tilde{R}}_{k-1}$, respectively, by Cholesky decomposition. Multiplying both sides of (23) on the left by ${(G}_{k-1}^{*}{)}^{-1}$, one obtains

$$O_{k-1}^{*}=W_{k-1}^{*}d_{k-1}+e_{k-1}^{*}$$

where $O_{k-1}^{*}={G_{k-1}^{*}}^{-1}\left[\begin{array}{c} {\bar{d}}_{k-1}\\ y_{k-1}-C_{k-1}{\hat{x}}_{k-1|k-2}^{*}-\varsigma_{k-1} \end{array}\right]$, $W_{k-1}^{*}={G_{k-1}^{*}}^{-1}\left[\begin{array}{c} I_{m}\\ D_{k-1} \end{array}\right]$, $e_{k-1}^{*}={G_{k-1}^{*}}^{-1}v_{k-1}^{*}$, ${e_{i}}_{,k-1}=o_{k-1}^{*i}-w_{k-1}^{*i}d_{k-1}$.

Subsequently, the constructed error is substituted into the second-order information potential objective function:

(26) $$J_{L1}\left(d_{k-1}\right)=\frac{1}{{N_{1}}^{2}}\sum_{i=1}^{N_{1}}\sum_{j=1}^{N_{1}}G_{\sigma_{1}}\left(e_{i}\left(k-1\right)-e_{j}\left(k-1\right)\right)$$

Equation (26) defines the second-order information potential of the constructed error and measures the similarity among the whitened error samples. A larger information potential indicates a more concentrated error distribution and, consequently, lower error entropy. Maximizing this objective therefore improves robustness against non-Gaussian noise and abnormal errors.

Although the whitened error components may not strictly satisfy the i.i.d. assumption, strict i.i.d. conditions are not required for constructing the vector-form MEE cost used in MEE-KF. Instead, these components are treated as covariance-normalized constructed error samples. Therefore, the lack of strict i.i.d. conditions mainly affects the probabilistic interpretation of the information potential but does not prevent the application of the MEE cost or alter the algebraic form of the fixed-point iteration. The numerical stability of the iteration mainly depends on bounded kernel weights, the positive definiteness of the equivalent weighted matrix, and an appropriate kernel bandwidth.

Since minimizing the error entropy is equivalent to maximizing the information potential, the unknown-input estimate can be obtained by maximizing the above objective function, i.e.,

(27) $${\hat{d}}_{k-1}=arg\;\underset{d_{k-1}}{\max}\frac{1}{{N_{1}}^{2}}\sum_{i=1}^{N_{1}}\sum_{j=1}^{N_{1}}G_{\sigma_{1}}\left(e_{i}\left(k-1\right)-e_{j}\left(k-1\right)\right)\;\;\;\;\;\;\;\;\;$$

here, $N_{1}=m+p$, By setting the gradient of the loss function $J_{L1}\left(d_{k-1}\right)$ with respect to ${\hat{d}}_{k-1}$ to zero, one obtains

$$\frac{\partial J_{L1}\left(d_{k-1}\right)}{\partial d_{k-1}}=0$$

Accordingly, the fixed-point iterative solution can be written as

(28) $${\hat{d}}_{k-1}^{\left(t+1\right)}=g_{1}\left({\hat{d}}_{k-1}^{\left(t\right)}\right)={\left({\left(W_{k-1}^{*}\right)}^{T}\Upsilon_{k-1}^{\left(t\right)}W_{k-1}^{*}\right)}^{-1}\left({\left(W_{k-1}^{*}\right)}^{T}\Upsilon_{k-1}^{\left(t\right)}O_{k-1}^{*}\right)$$

where

$$\Upsilon_{k-1}^{\left(t\right)}=\Omega_{k-1}^{\left(t\right)}-\Phi_{k-1}^{\left(t\right)}\;\Phi_{k-1}^{\left(t\right)}={\left[\varphi_{ij,k-1}^{\left(t\right)}\right]}_{N_{1}\times N_{1}}\;\Omega_{k-1}^{\left(t\right)}=diag(\sum_{j=1}^{N_{1}}\varphi_{1j,k-1}^{\left(t\right)},\ldots ,\sum_{j=1}^{N_{1}}\varphi_{N_{1}j,k-1}^{\left(t\right)})\;\varphi_{ij,k-1}^{\left(t\right)}=G_{\sigma_{1}}\left(e_{i}^{t}\left(k-1\right)-e_{j}^{t}\left(k-1\right)\right)\;\Upsilon_{k-1}^{\left(t\right)}=\left(\begin{array}{cc} \gamma_{d,k-1}^{\left(t\right)} & \gamma_{dr,k-1}^{\left(t\right)}\\ \gamma_{rd,k-1}^{\left(t\right)} & \gamma_{r,k-1}^{\left(t\right)} \end{array}\right)$$

then, $d_{k-1}$ can be solved by fixed-point iteration as

(29) $${\hat{d}}_{k-1}^{\left(t+1\right)}=M_{k-1}^{\left(t+1\right)}\left(y_{k-1}-C_{k-1}{\hat{x}}_{k-1|k-2}^{*}-\varsigma_{k-1}\right)$$

where t denotes the iteration index, and $M_{k-1}^{\left(t\right)}$ is the gain matrix corresponding to the t-th iteration, defined as

(30) $$M_{k-1}^{\left(t+1\right)}=\left(I_{m}-{\tilde{L}}_{k-1}^{\left(t\right)}D_{k-1}\right)M_{k-1}^{\left(t\right)}+{\tilde{L}}_{k-1}^{\left(t\right)}$$

$$\begin{array}{c} {\tilde{L}}_{k-1}^{\left(t\right)}={\left[{\tilde{P}}_{k-1}^{d,(t)}+D_{k-1}^{T}{\tilde{P}}_{dr,k-1}^{\left(t\right)}+\left(({\tilde{P}}_{rd,k-1}^{\left(t\right)}+D_{k-1}^{T}{\tilde{R}}_{k-1}^{*,(t)}{)D}_{k-1}\right)\right]}^{-1}\left({\tilde{P}}_{rd,k-1}^{\left(t\right)}+D_{k-1}^{T}{\tilde{R}}_{k-1}^{*,(t)}\right) \end{array}\;{\tilde{P}}_{k-1}^{d,(t)}={\left(G_{k-1}^{d}\right)}^{-T}\gamma_{d,k-1}^{\left(t\right)}{\left(G_{k-1}^{d}\right)}^{-1}\;{\tilde{P}}_{dr,k-1}^{\left(t\right)}={\left(G_{k-1}^{\tilde{r}}\right)}^{-T}\gamma_{dr,k-1}^{\left(t\right)}{\left(G_{k-1}^{d}\right)}^{-1}\;{\tilde{P}}_{rd,k-1}^{\left(t\right)}={\left(G_{k-1}^{d}\right)}^{-T}\gamma_{rd,k-1}^{\left(t\right)}{\left(G_{k-1}^{\tilde{r}}\right)}^{-1}\;{\tilde{R}}_{k-1}^{*,(t)}={\left(G_{k-1}^{\tilde{r}}\right)}^{-T}\gamma_{r,k-1}^{\left(t\right)}{\left(G_{k-1}^{\tilde{r}}\right)}^{-1}$$

where $M_{k-1}^{\left(t\right)}$ is the MEE-based unknown-input estimation gain at the t-th iteration. The iteration stops when

$$\frac{∥{\hat{d}}_{k-1}^{\left(t+1\right)}-{\hat{d}}_{k-1}^{\left(t\right)}∥}{∥{\hat{d}}_{k-1}^{\left(t\right)}∥}\leq \varepsilon_{d}$$

### 3.2. MEE State Estimation

For the given linear system model (5) and (6), the augmented pseudo-measurement model is established as follows:

(31) $$\left[\begin{array}{c} {\hat{x}}_{k-1|k-2}^{*}\\ y_{k-1}-\varsigma_{k-1} \end{array}\right]=\left[\begin{array}{c} I_{n}\\ C_{k-1} \end{array}\right]x_{k-1}+\left[\begin{array}{c} 0\\ D_{k-1} \end{array}\right]d_{k-1}+v_{k-1}$$

The corresponding noise term $V_{k-1}$ can be expressed as:

(32) $$v_{k-1}=\left[\begin{array}{c} -\left(x_{k-1}-{\hat{x}}_{k-1|k-2}^{*}\right)\\ r_{k-1} \end{array}\right]$$

where $x_{k-1}-{\hat{x}}_{k-1|k-2}^{*}$ denotes the local state prediction error, $r_{k-1}$ is the measurement noise, and $v_{k-1}$ is the augmented noise vector containing these two error terms. This compact form is introduced for constructing the subsequent whitened regression equation. According to (32), the covariance matrix of $v_{k-1}$ is given by

$$E\left[v_{k-1}v_{k-1}^{T}\right]=\left[\begin{array}{cc} P_{k-1|k-2}^{x*} & 0\\ 0 & R_{k-1} \end{array}\right]=\left[\begin{array}{cc} G_{k-1|k-2}^{P*}G_{k-1|k-2}^{P*T} & 0\\ 0 & G_{k-1}^{r}G_{k-1}^{rT} \end{array}\right]=G_{k-1}G_{k-1}^{T}$$

In the above equation $G_{k-1|k-2}^{P*}$ and $G_{k-1}^{r}$ are obtained from $P_{k-1|k-2}^{x*}$ and $R_{k-1}$, respectively, by Cholesky decomposition. By multiplying both sides of the equation by (${G_{k-1})}^{-1}$, one obtains:

(33) $$O_{k-1}=W_{k-1}^{1}x_{k-1}+w_{k-1}^{2}d_{k-1}+e_{k-1}$$

where

$$O_{k-1}=G_{k-1}^{-1}\left[\begin{array}{c} {\hat{x}}_{k-1|k-2}^{*}\\ y_{k-1}-\varsigma_{k-1} \end{array}\right],W_{k-1}^{1}=G_{k-1}^{-1}\left[\begin{array}{c} I_{n}\\ C_{k-1} \end{array}\right],W_{k-1}^{2}=G_{k-1}^{-1}\left[\begin{array}{c} 0\\ D_{k-1} \end{array}\right],e_{k-1}=G_{k-1}^{-1}v_{k-1}$$

Then, based on the minimum error entropy criterion, the following loss function is constructed:

(34) $$J_{L_{2}}\left(x_{k-1}\right)=\frac{1}{{N_{2}}^{2}}\sum_{i=1}^{N_{2}}\sum_{j=1}^{N_{2}}G_{\sigma_{2}}\left(e_{i,k-1}\left(x_{k-1}\right)-e_{j,k-1}\left(x_{k-1}\right)\right)$$

where $e_{i,k-1}\left(x_{k-1}\right)=\left(o_{k-1}^{i}-w_{k-1}^{1i}x_{k-1}-w_{k-1}^{2i}d_{k-1}\right),$ $N_{2}=n+p$, The optimal solution ${\hat{x}}_{k-1}^{*}$ obtained by maximizing the above loss function, i.e.,

(35) $${\hat{x}}_{k-1}^{*}=arg\underset{x_{k-1}}{max}\frac{1}{{N_{2}}^{2}}\sum_{i=1}^{N_{2}}\sum_{j=1}^{N_{2}}G_{\sigma_{2}}\left(e_{i,k-1}\left(x_{k-1}\right)-e_{j,k-1}\left(x_{k-1}\right)\right)$$

setting the gradient of the loss function $J_{L_{2}}\left(x_{k-1}\right)$ with respect to $x_{k-1}$ to zero, one obtains

$$\frac{\partial J_{L_{2}}\left(x_{k-1}\right)}{\partial x_{k-1}}=0$$

Thus, the fixed-point iterative solution can be written as

(36) $${\hat{x}}_{k-1}^{\left(t+1\right)}=g\left({\hat{x}}_{k-1}^{\left(t\right)}\right)={\left({\left(W_{k-1}^{1}\right)}^{T}\Upsilon_{k-1}^{1(t)}W_{k-1}^{1}\right)}^{-1}\left({\left(W_{k-1}^{1}\right)}^{T}\Upsilon_{k-1}^{1(t)}{(O}_{k-1}-W_{k-1}^{2}{\hat{d}}_{k-1}^{\left(t\right)})\right)$$

where

$$\Upsilon_{k-1}^{1(t)}=\Omega_{k-1}^{1(t)}-\Phi_{k-1}^{1(t)}\;\Phi_{k-1}^{1(t)}={\left[\varphi_{ij,k-1}^{1(t)}\right]}_{N_{2}\times N_{2}}\;\Omega_{k-1}^{1(t)}=diag(\omega_{1,k-1}^{1\left(t\right)},\ldots ,\omega_{N_{2},k-1}^{1(t)})\;\omega_{i,k-1}^{\left(t\right)}=\sum_{j=1}^{N_{2}}\varphi_{ij,k-1}^{\left(t\right)}\;\varphi_{ij,k-1}^{1\left(t\right)}=G_{\sigma 2}\left(e_{i}^{1,t}\left(k-1\right)-e_{j}^{1,t}\left(k-1\right)\right)\;\Upsilon_{k-1}^{1,(t)}=\left(\begin{array}{cc} \gamma_{x,k-1}^{1,(t)} & \gamma_{xr,k-1}^{1,(t)}\\ \gamma_{rx,k-1}^{1,(t)} & \gamma_{r,k-1}^{1,(t)} \end{array}\right)$$

Substituting the above definitions into (35) yields:

(37) $${{\hat{x}}^{*}}_{k-1|k-1}={\hat{x}}_{k-1|k-2}^{*}+{\tilde{K}}_{k-1}\left(y_{k-1}-C_{k-1}{\hat{x}}_{k-1|k-2}^{*}-D_{k-1}d_{k-1}^{\left(t\right)}-\varsigma_{k-1}\right)$$

where

$${\tilde{K}}_{k-1}^{\left(t\right)}={\left[{\tilde{P}}_{k-1|k-2}^{\left(t\right)}+C_{k-1}^{T}{\tilde{P}}_{xr,k-1|k-2}^{\left(t\right)}+\left({(\tilde{P}}_{rx,k-1|k-2}^{\left(t\right)}+C_{k-1}^{T}{\tilde{R}}_{k-1}^{\left(t\right)}{)C}_{k-1}\right)\right]}^{-1}\left({\tilde{P}}_{rx,k-1|k-2}^{\left(t\right)}+C_{k-1}^{T}{\tilde{R}}_{k-1}^{\left(t\right)}\right)\;{\tilde{P}}_{k-1|k-2}^{\left(t\right)}={\left(G_{k-1|k-2}^{P*}\right)}^{-T}\gamma_{x,k-1}^{1,(t)}{\left(G_{k-1|k-2}^{P*}\right)}^{-1}\;{\tilde{P}}_{xr,k-1|k-2}^{\left(t\right)}={\left(G_{k-1}^{r}\right)}^{-T}\gamma_{xr,k-1}^{1,(t)}{\left(G_{k-1|k-2}^{P*}\right)}^{-1}\;{\tilde{P}}_{rx,k-1|k-2}^{\left(t\right)}={\left(G_{k-1|k-2}^{P*}\right)}^{-T}\gamma_{rx,k-1}^{1,(t)}{\left(G_{k-1}^{r}\right)}^{-1}\;{\tilde{R}}_{k-1}={\left(G_{k-1}^{r}\right)}^{-T}\gamma_{r,k-1}^{1,(t)}{\left(G_{k-1}^{r}\right)}^{-1}$$

where $K_{k-1}^{\left(t\right)}$ is the MEE-based state correction gain at the t-th iteration. The iteration stops when

$$\frac{∥{\hat{x}}_{k-1|k-1}^{\left(t+1\right)}-{\hat{x}}_{k-1|k-1}^{\left(t\right)}∥}{∥{\hat{x}}_{k-1|k-1}^{\left(t\right)}∥}\leq \varepsilon_{x}$$

**Remark 2.**  *The convergence behavior of the above MEE-based fixed-point iteration can be discussed similarly to that of the MEEKF [20]. After covariance-based whitening, the error components have a unified scale, and the Gaussian-kernel weights are bounded. Selecting a proper kernel bandwidth and keeping the covariance matrices positive can help maintain the numerical stability of the iteration.*

In the above expressions $d_{k-1}^{\left(t\right)}$ is the unknown input estimated in the first step. Accordingly, the corresponding estimation error can be written as

(38) $$\begin{array}{c} {\tilde{x}}_{k-1|k-1}^{\left(t\right)}=\left[I_{n}+{\tilde{K}}_{k-1}^{\left(t\right)}\left(D_{k-1}M_{k-1}^{\left(t\right)}-I_{p}\right)C_{k-1}\right]{\tilde{x}}_{k-1|k-2}^{*}\\ +{\tilde{K}}_{k-1}^{\left(t\right)}\left(D_{k-1}M_{k-1}^{\left(t\right)}-I_{p}\right){\bar{r}}_{k-1} \end{array}$$

(39) $$\begin{array}{c} P_{k-1|k-1}^{(t)x*}=\left[I_{n}+{\tilde{K}}_{k-1}^{\left(t\right)}\left(D_{k-1}M_{k-1}^{\left(t\right)}-I_{p}\right)C_{k-1}\right]P_{k-1|k-2}^{x*}{\left[I_{n}+{\tilde{K}}_{k-1}^{\left(t\right)}\left(D_{k-1}M_{k-1}^{\left(t\right)}-I_{p}\right)C_{k-1}\right]}^{T}\\ +\;{\tilde{K}}_{k-1}^{\left(t\right)}\left(D_{k-1}M_{k-1}^{\left(t\right)}-I_{p}\right){\tilde{R}}_{k-1}{\left(D_{k-1}M_{k-1}^{\left(t\right)}-I_{p}\right)}^{T}{{\tilde{K}}_{k-1}^{\left(t\right)}}^{T}\; \end{array}$$

### 3.3. Time Update

Based on the unknown input estimate ${\hat{d}}_{k-1}^{*}$ obtained in Section 3.1 and the state update estimate ${\hat{x}}_{k-1|k-1}^{*}$ obtained in Section 3.2, the one-step state prediction can be expressed as

(40) $${\hat{x}}_{k|k-1}^{*}=A_{k-1}{{\hat{x}}^{*}}_{k-1|k-1}+B_{k-1}{\hat{d}}_{k-1}^{*}+\xi_{k-1}$$

(41) $$P_{k|k-1}^{x*}=\left[\begin{array}{cc} A_{k-1} & B_{k-1} \end{array}\right]\left[\begin{array}{cc} P_{k-1|k-1}^{x*} & P_{k-1|k-1}^{xd*}\\ P_{k-1|k-1}^{dx*} & P_{k-1|k-1}^{d*} \end{array}\right]\left[\begin{array}{c} A_{k-1}^{T}\\ B_{k-1}^{T} \end{array}\right]+Q_{k-1}$$

When the unknown input estimate satisfies the unbiasedness condition $M_{k-1}^{*}D_{k-1}=I_{m}$, the cross covariance $P_{k-1|k-1}^{xd*}$ can be expressed as

(42) $$\begin{array}{l} P_{k-1|k-1}^{xd*}={\left(P_{k-1|k-1}^{dx*}\right)}^{T}=E\left[{\tilde{x}}_{k-1|k-1}^{*}{{\tilde{d}}_{k-1}^{*}}^{T}\right]\\ \;\;\;\;\;\;=-\left[I_{n}+{\tilde{K}}_{k-1}^{*}\left(D_{k-1}M_{k-1}^{*}-I_{p}\right)C_{k-1}\right]P_{k-1|k-2}^{x*}C_{k-1}^{T}M_{k-1}^{*T}-{\tilde{K}}_{k-1}^{*}\left(D_{k-1}M_{k-1}^{*}-I_{p}\right)R_{k-1}M_{k-1}^{*T} \end{array}$$

### 3.4. Multi-Model MEERTSF

The MEERTSF derived above is based on a locally linearized nonlinear model. For strongly nonlinear systems, a single expansion point may produce large linearization errors, which can further degrade unknown-input estimation and state correction. To reduce this effect, a multi-model MEERTSF is developed. The proposed method constructs multiple local linear models in the state uncertainty region. First, $N_{s}$ prediction expansion points are generated around the posterior estimate at time k−2. Then, $N_{s}$ measurement expansion points are generated under each prediction branch. Thus, the total number of joint branches is $B=N_{s}^{2}$. Each branch independently performs the MEERTSF recursion, and the final estimate is obtained by compatibility-based posterior fusion. The expansion points are generated only in the state space, and no prior probability model is assumed for the unknown input. Consider

$$x_{k-1}=f\left(x_{k-2},d_{k-2}\right)+q_{k-2},\;y_{k-1}=h(x_{k-1},d_{k-1})+r_{k-1}.$$

In this paper, the nonlinear simulation is conducted for a scalar nonlinear system. Therefore, the mass points are generated in the one-dimensional state space. Let the fused posterior estimate and covariance at time k−2 be ${\hat{x}}_{k-2|k-2}$ and $P_{k-2|k-2}$. The square-root factor is

$$S_{k-2}=\sqrt{P_{k-2|k-2}}$$

The normalized mass points are selected by the equal-probability rule:

$$l_{i}=\Phi^{-1}\left(\frac{i-0.5}{N_{s}}\right),\omega_{i}=\frac{1}{N_{s}},i=1,2,\ldots ,N_{s}$$

where $\Phi^{-1}(\cdot )$ is the inverse standard Gaussian cumulative distribution function. The prediction expansion points are

$$\chi_{k-2}^{i}={\hat{x}}_{k-2|k-2}+S_{k-2}l_{i}\;N_{s}=3,l=[-0.9674,0,0.9674]\;N_{s}=5,l=[-1.2816,-0.5244,0,0.5244,1.2816]$$

This scalar rule makes the nonlinear simulation directly reproducible. Multidimensional point generation can be realized by tensor-product quadrature or sigma-point rules, but it is not the focus of this paper.

For the i-th prediction point, the state equation is linearized as

$$x_{k-1}\approx A_{k-2}^{i}x_{k-2}+B_{k-2}^{i}d_{k-2}+\xi_{k-2}^{i}+q_{k-2}$$

where

$$\begin{array}{l} A_{k-2}^{i}={\frac{\partial f(x,d)}{\partial x}|}_{\left(\chi_{k-2}^{i},{\hat{d}}_{k-2}\right)},B_{k-2}^{i}={\frac{\partial f(x,d)}{\partial d}|}_{\left(\chi_{k-2}^{i},{\hat{d}}_{k-2}\right)},\\ \xi_{k-2}^{i}=f(\chi_{k-2}^{i},{\hat{d}}_{k-2})-A_{k-2}^{i}\chi_{k-2}^{i}-B_{k-2}^{i}{\hat{d}}_{k-2}. \end{array}$$

Here, $\xi_{k-2}^{i}$ is the affine compensation term. The predicted state and covariance are

$$\begin{array}{l} {\hat{x}}_{k-1|k-2}^{i}=A_{k-2}^{i}{\hat{x}}_{k-2|k-2}+B_{k-2}^{i}{\hat{d}}_{k-2}+\xi_{k-2}^{i},\\ P_{k-1|k-2}^{i}=A_{k-2}^{i}P_{k-2|k-2}(A_{k-2}^{i}{)}^{T}+Q_{k-2}. \end{array}$$

For the i-th prediction branch, let

$$P_{k-1|k-2}^{i}=S_{k-1|k-2}^{i}(S_{k-1|k-2}^{i}{)}^{T}.$$

The measurement expansion points are

$$\chi_{k-1}^{ij}={\hat{x}}_{k-1|k-2}^{i}+S_{k-1|k-2}^{i}\gamma_{j},j=1,2,\ldots ,N_{s}$$

For the $\left(i,j\right)$-th branch, the measurement equation is linearized as

$$y_{k-1}\approx C_{k-1}^{ij}x_{k-1}+D_{k-1}^{ij}d_{k-1}+\zeta_{k-1}^{ij}+r_{k-1}$$

where

$$\begin{array}{l} C_{k-1}^{ij}={\frac{\partial h(x,d)}{\partial x}|}_{\left(\chi_{k-1}^{ij},{\bar{d}}_{k-1}^{ij}\right)},D_{k-1}^{ij}={\frac{\partial h(x,d)}{\partial d}|}_{\left(\chi_{k-1}^{ij},{\bar{d}}_{k-1}^{ij}\right)},\\ \zeta_{k-1}^{ij}=h(\chi_{k-1}^{ij},{\bar{d}}_{k-1}^{ij})-C_{k-1}^{ij}\chi_{k-1}^{ij}-D_{k-1}^{ij}{\bar{d}}_{k-1}^{ij} \end{array}$$

Moving the affine term to the left-hand side gives

$${\tilde{y}}_{k-1}^{ij}=y_{k-1}-\zeta_{k-1}^{ij}$$

and the equivalent local measurement model becomes

$${\tilde{y}}_{k-1}^{ij}=C_{k-1}^{ij}x_{k-1}+D_{k-1}^{ij}d_{k-1}+r_{k-1}$$

For each joint branch, the MEERTSF recursion is applied to the equivalent local model. The unknown input and state are updated as

$$\begin{array}{l} {\hat{d}}_{k-1}^{ij}=M_{k-1}^{ij}\left({\tilde{y}}_{k-1}^{ij}-C_{k-1}^{ij}{\hat{x}}_{k-1|k-2}^{i}\right),\\ {\hat{x}}_{k-1|k-1}^{ij}={\hat{x}}_{k-1|k-2}^{i}+K_{k-1}^{ij}\left({\tilde{y}}_{k-1}^{ij}-C_{k-1}^{ij}{\hat{x}}_{k-1|k-2}^{i}-D_{k-1}^{ij}{\hat{d}}_{k-1}^{ij}\right), \end{array}$$

where $M_{k-1}^{ij}$ and $K_{k-1}^{ij}$ are obtained by the MEE fixed-point iterations. The corresponding branch result is

$$\left\{{\hat{x}}_{k-1|k-1}^{ij},{\hat{d}}_{k-1}^{ij},P_{k-1|k-1}^{ij}\right\}$$

After all $B=N_{s}^{2}$ branches are updated, the prior weight, predicted measurement, residual, and residual covariance of the $\left(i,j\right)$-th branch are defined as

$$\begin{array}{l} \pi_{k-1}^{ij}=\omega_{i}\omega_{j},\\ {\hat{y}}_{k-1}^{ij}=C_{k-1}^{ij}{\hat{x}}_{k-1|k-2}^{i}+D_{k-1}^{ij}{\hat{d}}_{k-1}^{ij}+\zeta_{k-1}^{ij},\\ \nu_{k-1}^{ij}=y_{k-1}-{\hat{y}}_{k-1}^{ij},\\ S_{k-1}^{ij}=C_{k-1}^{ij}P_{k-1|k-2}^{i}(C_{k-1}^{ij}{)}^{T}+R_{k-1}. \end{array}$$

Since the measurement noise is non-Gaussian, the following term is used only as a Gaussian-form compatibility index rather than an exact likelihood:

(43) $$\beta_{k-1}^{ij}=\frac{1}{\left(2\pi {)}^{p/2}{|S_{k-1}^{ij}|}^{1/2}\right.}\exp\left[-\frac{1}{2}(\nu_{k-1}^{ij}{)}^{T}(S_{k-1}^{ij}{)}^{-1}\nu_{k-1}^{ij}\right]$$

The posterior fusion weight is

(44) $$\mu_{k-1}^{ij}=\frac{\pi_{k-1}^{ij}\beta_{k-1}^{ij}}{\sum_{i=1}^{N_{s}}\sum_{j=1}^{N_{s}}{\pi_{k-1}^{ij}\beta }_{k-1}^{ij}},\sum_{i=1}^{N_{s}}\sum_{j=1}^{N_{s}}\mu_{k-1}^{ij}=1$$

The fused estimates are

(45) $${\hat{x}}_{k-1|k-1}=\sum_{i=1}^{N_{s}}\sum_{j=1}^{N_{s}}\mu_{k-1}^{ij}{\hat{x}}_{k-1|k-1}^{ij}$$

(46) $${\hat{d}}_{k-1}=\sum_{i=1}^{N_{s}}\sum_{j=1}^{N_{s}}\mu_{k-1}^{ij}{\hat{d}}_{k-1}^{ij}$$

(47) $$P_{k-1|k-1}=\sum_{i=1}^{N_{s}}\sum_{j=1}^{N_{s}}\mu_{k-1}^{ij}\left[P_{k-1|k-1}^{ij}+({\hat{x}}_{k-1|k-1}^{ij}-{\hat{x}}_{k-1|k-1}){\left({\hat{x}}_{k-1|k-1}^{ij}-{\hat{x}}_{k-1|k-1}\right)}^{T}\right]$$

In summary, the proposed method constructs $B=N_{s}^{2}$ local branches by a two-layer scalar mass-point expansion. Each branch performs an MEERTSF update, and the final estimate is obtained through compatibility-based posterior fusion. This structure reduces the linearization error of single-point MEERTSF while retaining the robustness of the MEE criterion under non-Gaussian noise.

## 4. Computational Complexity

This section analyzes the dominant computational complexity of the proposed MMMEERTSF at each sampling instant. Let n, m, and p denote the dimensions of the state vector, the unknown-input vector, and the measurement vector, respectively. In the MEE-based unknown-input estimation step, the augmented residual dimension is $L_{d}=m+p$. In the MEE-based state-correction step, the augmented residual dimension is $L_{x}=n+p$. Let $T_{d}$ and $T_{x}$ denote the average numbers of fixed-point iterations required for unknown-input estimation and state correction, respectively.

For the single-model MEERTSF, the main computational burden comes from the construction of the MEE weighting matrix and the computation of the equivalent gain matrices. Since the MEE criterion uses pairwise error differences, the weighting matrix construction has a quadratic dependence on the residual dimension. Thus, the dominant cost of one fixed-point iteration in the unknown-input estimation step is

$$O(L_{d}^{2}m+m^{3}+p^{3})$$

where $L_{d}^{2}m$ is mainly related to entropy-weighted matrix multiplication, $m^{3}$ comes from the inversion of the unknown-input-related matrix, and $p^{3}$ is associated with measurement covariance operations. Similarly, the dominant cost of one fixed-point iteration in the state-correction step is

$$O(L_{x}^{2}n+n^{3}+p^{3})$$

Therefore, the dominant complexity of the single-model MEERTSF at one sampling instant is

$$C_{MEERTSF}=O\left[T_{d}(L_{d}^{2}m+m^{3}+p^{3})+T_{x}(L_{x}^{2}n+n^{3}+p^{3})\right]$$

In the proposed MMMEERTSF, $N_{s}$ prediction-layer expansion points and $N_{s}$ measurement-layer expansion points are used. Hence, the total number of joint local branches is $B=N_{s}^{2}$. Since each branch performs one complete MEERTSF recursion, the dominant complexity of MMMEERTSF is approximately

$$C_{MMMEERTSF}=O\left\{N_{s}^{2}\left[T_{d}(L_{d}^{2}m+m^{3}+p^{3})+T_{x}(L_{x}^{2}n+n^{3}+p^{3})\right]\right\}$$

Additional operations, including local linearization, residual compatibility evaluation, and weighted fusion, also introduce extra computational costs. However, these operations are usually lower than the repeated MEE fixed-point iterations over all $N_{s}^{2}$ branches and are therefore not included in the dominant complexity expression.

In summary, the computational complexity of MMMEERTSF increases approximately linearly with the number of joint branches $N_{s}^{2}$. Compared with single-model MEERTSF, the additional burden mainly comes from applying the MEE-based unknown-input estimation and state-correction steps to multiple local branches. When $N_{s}$, $T_{d}$, and $T_{x}$ are properly selected, the computational cost remains acceptable while the multi-model structure improves the approximation accuracy for strongly nonlinear systems.

## 5. Simulation Experiment Comparison

In this section, two simulation experiments are provided to demonstrate the performance of MEERTSF in a linear environment and MM-MEERTSF in a nonlinear environment, respectively. Gaussian mixture noise and outlier-contaminated noise are adopted to evaluate the robustness of the proposed algorithms under non-Gaussian disturbances.

### 5.1. Land-Vehicle State Estimation Under Non-Gaussian Noise

The performance of RTSF, HuberRTSF, MCRTSF and MEERTSF is evaluated in a land-vehicle navigation scenario under non-Gaussian measurement noise. A slightly modified land-vehicle navigation model with an unknown input is considered as follows:

$$\begin{array}{l} x_{k}=\left[\begin{array}{cccc} 1 & 0 & \Delta T & 0\\ 0 & 1 & 0 & \Delta T\\ 0 & 0 & 1 & 0\\ 0 & 0 & 0 & 1 \end{array}\right]x_{k-1}+\left[\begin{array}{c} 0.04\\ 0.01\\ 0.05\\ 0.02 \end{array}\right]d_{k-1}+q_{k-1}\\ y_{k-1}^{\left(j\right)}=\left[\begin{array}{cccc} -1 & 0 & -1 & 0\\ 0 & -1 & 0 & -1 \end{array}\right]x_{k-1}+\left[\begin{array}{c} \phi_{2j-1}\\ \phi_{2j} \end{array}\right]d_{k-1}+r_{k-1}^{\left(j\right)},j=1,2,3,4 \end{array}$$

where $x_{k-1}=[x_{1,k-1},x_{2,k-1},x_{3,k-1},x_{4,k-1}{]}^{T}$ denotes the vehicle state vector. The components $x_{1,k}$ and $x_{2,k}$ represent the north and east positions, while $x_{3,k}$ and $x_{4,k}$ represent the northward and eastward velocities, respectively. The parameters ΔT=0.3 and θ=π/3 denote the sampling interval and the vehicle heading angle, respectively. In the measurement equation, $y_{k-1}^{\left(j\right)}\in R^{2}$ denotes the output of the j-th measurement group. To increase the measurement redundancy and improve the observability of the system, four groups of two-dimensional measurement channels are used in the simulation. Therefore, the complete measurement vector is obtained by stacking the four groups as

$$y_{k-1}={\left[\begin{array}{cccc} {\left(y_{k-1}^{\left(1\right)}\right)}^{T} & {\left(y_{k-1}^{\left(2\right)}\right)}^{T} & {\left(y_{k-1}^{\left(3\right)}\right)}^{T} & {\left(y_{k-1}^{\left(4\right)}\right)}^{T} \end{array}\right]}^{T}\in R^{8}$$

The corresponding unknown-input direct feedthrough coefficients are selected as

$$\Phi ={\left[\begin{array}{cccccccc} 1.0 & -2.8 & 2.5 & -0.8 & -1.2 & 3.0 & 0.6 & 1.5 \end{array}\right]}^{T}$$

The process noise $q_{k}$ is assumed to be zero-mean Gaussian noise with covariance $Q=0.01I_{4}$. The unknown input $d_{k-1}$ is set as a composite signal consisting of a sinusoidal component and Gaussian white noise, namely $d_{k-1}=\sin\left(\frac{2\pi \cdot 10(k-1)}{N}\right)+0.2\omega_{k-1}$, where $\omega_{k-1}∼N\left(0,1\right)$. This setting is used to describe a time-varying unknown external input acting on the vehicle system. To evaluate the robustness of the proposed method under non-Gaussian disturbances, the measurement noise is generated by a three-component Gaussian mixture distribution: $r_{k-1}∼0.48N\left(-0.1,0.001\right)+0.04N\left(0,1000\right)+0.48N\left(0.1,0.001\right).$ This noise distribution has a clear multi-peak characteristic and contains a small proportion of large-amplitude impulsive noise. Hence, it can be used to examine the robustness of different filters against non-Gaussian measurement disturbances. In the filters, the nominal measurement noise covariance is set according to the variance of the above mixture noise. The initial true state is set as $x_{0}=[0,0,10tan\theta ,10{]}^{T}$, and the prior estimate is initialized as ${\hat{x}}_{0|0}=[1,1,1,1{]}^{T}$. The prior error covariance is initialized as $P_{0|0}=diag\left\{900,900,4,4\right\}.$ The simulation length is N=500, and 100 independent Monte Carlo runs are performed. For the final comparison results, the RMSE values are reported as mean ± standard deviation over these Monte Carlo runs. To further compare different robust criteria within the RTSF framework, HuberRTSF is introduced as an additional comparison method. In HuberRTSF, the Huber loss is used to reweight the whitened residuals in both the unknown-input estimation and state-correction steps. When the residual is small, the corresponding weight is close to that of the conventional quadratic criterion. When the residual exceeds the threshold δ, its weight is reduced to weaken the influence of outliers. Here, δ denotes the Huber threshold. According to the parameter-tuning results in Table 1, the threshold of HuberRTSF is set to δ=0.5, the kernel width of MCRTSF is set to $\sigma_{1}=3$, and the kernel width of MEERTSF is set to $\sigma_{1}=1$. The maximum number of fixed-point iterations is set to 50, and the convergence threshold is $10^{-6}$. The root mean square error obtained from the Monte Carlo runs is adopted as the performance index.

**Figure 1:**
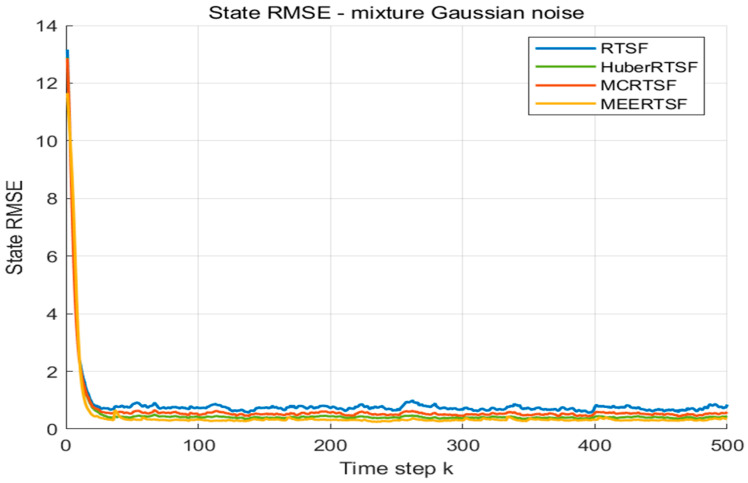
Overall state RMSE comparison among RTSF, MCRTSF, HuberRTSF and MEERTSF.

Figure 1 illustrates the comparison of state RMSE for RTSF, HuberRTSF, MCRTSF, and MEERTSF under multimodal non-Gaussian noise. Due to biases in the initial estimates, all algorithms exhibit large RMSE values in the initial stage, which then rapidly decrease and stabilize. Compared with RTSF, both HuberRTSF and MCRTSF can reduce the estimation error to a certain extent. Among them, MEERTSF achieves the lowest state RMSE in the steady-state stage, indicating that the RTSF under the MEE criterion is more robust to multimodal non-Gaussian noise.

**Figure 2:**
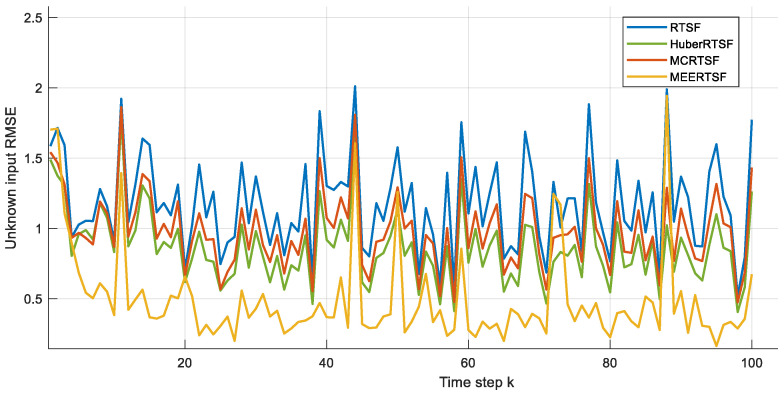
Unknown-input RMSE comparison among RTSF, MCRTSF, HuberRTSF and MEERTSF.

Figure 2 presents the comparison of unknown input RMSE for RTSF, HuberRTSF, MCRTSF, and MEERTSF under multimodal non-Gaussian noise with outliers. It can be observed that the unknown input estimation is more sensitive to outlier interference, and the RMSE of each algorithm exhibits certain fluctuations. Compared with RTSF, HuberRTSF and MCRTSF can suppress some spike errors, indicating that the robust criteria have a certain mitigating effect on abnormal noise. However, MEERTSF achieves the lowest overall RMSE with smaller fluctuations, demonstrating its superior robustness and accuracy in unknown input estimation.

**Figure 3:**
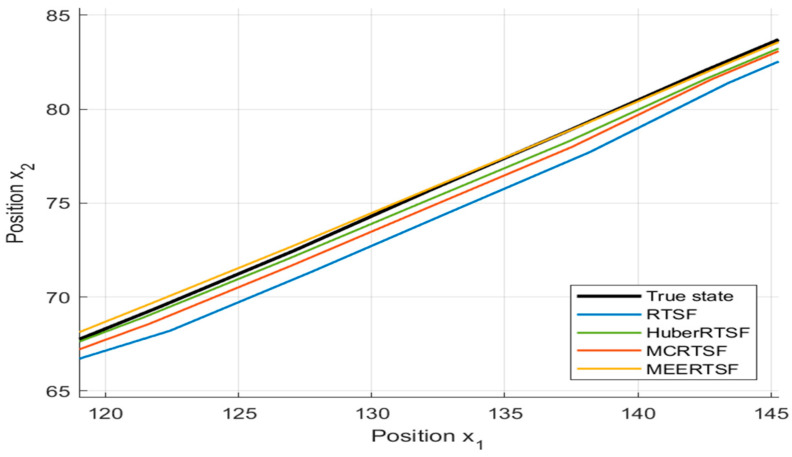
Comparison of estimated and true trajectories.

Figure 3 provides a zoomed-in comparison of the true state trajectory and the estimated trajectories from different algorithms. As the full trajectory is too long to clearly reveal estimation error differences, a local segment is shown. All algorithms track the true trajectory reasonably well. HuberRTSF, MCRTSF, and MEERTSF follow the true state more closely, with MEERTSF achieving the closest agreement, further confirming its superior state estimation accuracy under non-Gaussian noise.

Table 1 presents the parameter sensitivity analysis of HuberRTSF, MCRTSF, and MEERTSF under Gaussian mixture noise, which is used to select the parameters for the following comparison experiments, listing the ARMSEs of RTSF, HuberRTSF, MCRTSF, and MEERTSF under Gaussian mixture noise. The variables $x_{1}$, $x_{2}$, $x_{3}$, and $x_{4}$ denote the four state components, and d denotes the unknown input. A smaller ARMSE indicates higher estimation accuracy. For HuberRTSF, the threshold parameter δ has a clear influence on the estimation performance, and δ=0.5 provides a better overall result. As δ increases, the suppression effect on large residuals becomes weaker, which leads to larger estimation errors under non-Gaussian noise. For MCRTSF and MEERTSF, the kernel width $\sigma_{1}$ is an important parameter affecting the robustness of the filters. MCRTSF achieves better overall performance around $\sigma_{1}=3$. For MEERTSF, $\sigma_{1}=1.0$ gives better results for $x_{1}$, $x_{2}$, and the unknown input d, especially in reducing the unknown-input estimation error. It should be noted that different robust criteria show different behaviors for different state components. MEERTSF does not obtain the minimum ARMSE for all state components, but it provides a good balance between state estimation and unknown-input estimation. Therefore, δ=0.5, $\sigma_{1}=3$, and $\sigma_{1}=1$. are selected as the parameter settings of HuberRTSF, MCRTSF, and MEERTSF in the following simulations, respectively.

Table 2 reports the RMSEs of RTSF, HuberRTSF, MCRTSF, and MEERTSF under different noise conditions. Under Gaussian noise, all filters achieve similar RMSEs, indicating that the conventional minimum-variance criterion still works well in near-Gaussian environments, and the advantage of robust criteria is not significant in this case. When outliers or Gaussian mixture noise are introduced, the robust filters generally outperform RTSF, which shows that the Huber, MCC, and MEE criteria can suppress the influence of non-Gaussian noise to different extents. Under Gaussian mixture noise and Gaussian mixture noise with outliers, MEERTSF obtains lower RMSEs for $x_{1}$, $x_{2}$, and the unknown input d, especially in unknown-input estimation. Meanwhile, HuberRTSF and MCRTSF also provide competitive results for some state components, indicating that different robust criteria have different sensitivities to the noise type and state component. Overall, MEERTSF shows better comprehensive estimation performance under complex non-Gaussian noise. This advantage is mainly attributed to the minimum error entropy criterion, which exploits the overall distribution information of the estimation error rather than only minimizing the second-order moment. Therefore, MEERTSF can produce a more concentrated error distribution and improve the robustness of the filter under multi-peak non-Gaussian measurement noise.

Overall, under Gaussian mixture noise, MEERTSF provides better comprehensive performance, particularly in unknown-input estimation, while HuberRTSF and MCRTSF remain competitive for some state components. This improvement is mainly attributed to the minimum error entropy criterion, which considers the overall distribution of the estimation error rather than only minimizing the second-order moment. Therefore, MEERTSF can obtain a more concentrated error distribution and achieve better robustness under multi-peak non-Gaussian measurement noise.

### 5.2. Multi-Model MEERTSF Simulation Experiment

To evaluate the performance of the proposed multi-model MEERTSF under nonlinear conditions, a scalar nonlinear system with unknown input is considered:

$$x_{k}=0.5x_{k-1}+\frac{25x_{k-1}}{1+x_{k-1}^{2}}+8cos[1.2(k-1)]+Gd_{k-1}+q_{k-1}$$

where $x_{k}\in R^{1}$ is the system state, $d_{k-1}$ is the unknown input, and $q_{k-1}$ is the process noise. The unknown-input coefficient is set as G=0.5.

The nonlinear measurement equation is given in a two-dimensional group form:

$$y_{k-1}^{\left(j\right)}=\left[\begin{array}{c} c_{2j-1}\\ c_{2j} \end{array}\right]\frac{x_{k-1}^{2}}{20}+\left[\begin{array}{c} \phi_{2j-1}\\ \phi_{2j} \end{array}\right]d_{k-1}+r_{k-1}^{\left(j\right)},j=1,2,3,4.$$

The complete measurement vector is obtained by stacking four two-dimensional measurement groups:

$$y_{k-1}={\left[\begin{array}{cccc} {\left(y_{k-1}^{\left(1\right)}\right)}^{T} & {\left(y_{k-1}^{\left(2\right)}\right)}^{T} & {\left(y_{k-1}^{\left(3\right)}\right)}^{T} & {\left(y_{k-1}^{\left(4\right)}\right)}^{T} \end{array}\right]}^{T}\in R^{8}$$

The sensor coefficients and the unknown-input direct feedthrough vector are selected as

$$\begin{array}{l} C=[1.00,0.90,1.10,0.80,1.20,0.70,1.30,0.95{]}^{T},\\ \Phi =[1.0,-2.8,2.5,-0.8,-1.2,3.0,0.6,1.5{]}^{T}. \end{array}$$

The process noise is zero-mean Gaussian noise with variance Q=1. The unknown input is generated as $d_{k-1}=sin\left(\frac{2\pi \cdot 10(k-1)}{N}\right)+0.2\omega_{k-1},\omega_{k-1}∼N(0,1)$. The measurement noise follows a three-component Gaussian mixture distribution:

$$r_{k-1}∼\left\{\begin{array}{cc} N(-0.1,0.001), & p=0.48,\\ N(0,1000), & p=0.04,\\ N(0.1,0.001), & p=0.48. \end{array}\right.$$

This distribution has a clear multi-peak characteristic and contains impulsive disturbances. In the filtering process, the nominal measurement noise covariance is set according to the equivalent variance of the mixture noise $R=40.01056I_{8}$, the initial true state, initial estimate, and initial covariance are set as $x_{0}=0.1,{\hat{x}}_{0|0}=0.2,P_{0|0}=2$. The initial unknown-input estimate is ${\hat{d}}_{0}=0$. The auxiliary covariance for the unknown-input residual is initialized as $P_{0}^{d}=\rho_{d}I_{m}$, where $\rho_{d}=1$. This parameter is only used for residual whitening and weighting in the fixed-point iteration.

The simulation length is N=100, and 100 independent Monte Carlo runs are performed. Since the simulation model is changed from the linear case to the nonlinear multi-model case, a small-range parameter retuning is required. Following the same parameter-sensitivity tuning procedure as in the linear simulation, the parameters for the nonlinear simulation were selected as $\delta_{d}=\delta_{x}=0.4$ for Multi-model HuberRTSF, $\sigma_{d}=\sigma_{x}=8$ for Multi-model MCRTSF, and $\sigma_{d}=\sigma_{x}=4$ for Multi-model MEERTSF. These settings provided the best overall estimation performance among the tested values.

The maximum number of fixed-point iterations is 50, and the convergence threshold is $10^{-6}$. The compared algorithms are Multi-model HuberRTSF, Multi-model MCRTSF, and Multi-model MEERTSF. All three filters use the same three-point multi-model structure, with

l={−0.9674,0,0.9674}

which produces 3×3=9 local branches. The prior weights of the sampling points are equal. After each branch completes the MEERTSF update, the posterior branch weights are calculated using the Gaussian-form residual compatibility index, and the final estimates are obtained by weighted fusion. The RMSE over 100 Monte Carlo runs is used as the performance index. This experiment aims to verify whether the multi-model expansion can improve MEERTSF by reducing the linearization error under strongly nonlinear and non-Gaussian conditions.

**Figure 4:**
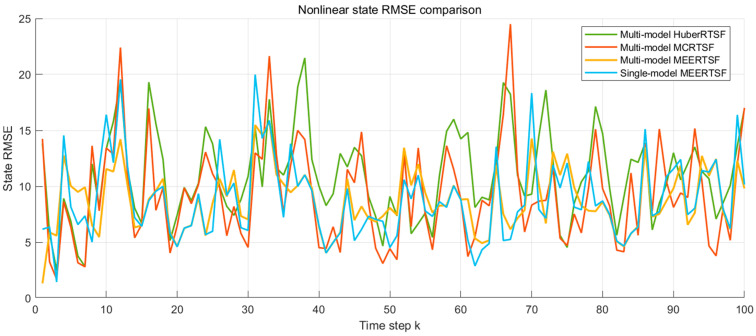
State RMSE comparison among Single-model MEERTSF, Multi-model HuberRTSF, Multi-model MCRTSF, and Multi-model MEERTSF.

Figure 4 compares the state RMSEs of Single-model MEERTSF, Multi-model HuberRTSF, Multi-model MCRTSF, and Multi-model MEERTSF for the nonlinear model. The RMSE curves fluctuate due to nonlinear dynamics and non-Gaussian noise. Compared with Single-model MEERTSF and the other multi-model robust filters, Multi-model MEERTSF generally achieves lower RMSE and better suppresses large error fluctuations, demonstrating its improved robustness and estimation accuracy.

## 6. Conclusions

This paper has addressed the problem of estimation bias and abrupt error growth in nonlinear systems under non-Gaussian heavy-tailed noise and impulsive interference. By exploiting the structural advantages of the recursive three-step filter (RTSF), a robust nonlinear state estimation method has been developed by integrating the minimum error entropy (MEE) criterion with a multi-model strategy. The main conclusions are summarized as follows.

(1) The MEE criterion has been introduced into the three-step recursive framework of RTSF, namely, unknown-input estimation, measurement update, and time update. By incorporating kernel density estimation into the error characterization and adopting an iterative reweighting mechanism, the proposed method can adaptively suppress abnormal residuals, thereby improving the robustness and stability of the filter under heavy-tailed noise. At the same time, the recursive structure and computational implementability of RTSF are preserved, making the algorithm suitable for online estimation.
(2) To mitigate the performance degradation caused by linearization errors and insufficient local approximation in nonlinear models, a multi-model/multi-point approximation strategy has been further incorporated. By using a finite number of representative points to cover the nonlinear mappings locally, the proposed method performs local updates on different model branches and then fuses the corresponding estimates, thereby effectively reducing the bias accumulation caused by single-point linearization. Simulation results have shown that, in scenarios with strong nonlinearity and heavy-tailed noise, the proposed MM-MEERTSF achieves lower RMSE than the single-point MEERTSF under the nonlinear simulation scenario, while also suppressing error spikes caused by abnormal measurements to a certain extent.
(3) From the perspective of engineering implementation and performance consistency, this paper has also examined the influence of parameter selection on filtering performance and provided a recommended guideline that balances robustness and convergence efficiency. In addition, the updating strategy of the multi-model fusion weights can provide more stable estimation outputs when model uncertainty becomes significant, which offers support for future applications in complex-noise and strongly nonlinear scenarios.

Although the proposed MMMEERTSF improves robustness and estimation accuracy under non-Gaussian noise, several issues remain to be further investigated. These include the computational burden caused by MEE-based fixed-point iterations and the multi-model structure, the coupling between unknown-input estimation and state correction, the adaptive selection of kernel bandwidths, and the improvement of nonlinear multi-model strategies. Future work will focus on low-complexity implementation, adaptive parameter selection, and validation using practical engineering data.

In summary, the proposed multi-model minimum error entropy recursive three-step filter enhances robustness against non-Gaussian heavy-tailed noise and abnormal disturbances while preserving the recursive structure and online computational feasibility. It also achieves superior overall performance in nonlinear state estimation.

## Figures and Tables

**Table 1:** Parameter sensitivity analysis under Gaussian mixture noise.

ARMSE of Filters	$x_{1}$	$x_{2}$	$x_{3}$	$x_{4}$	d
RTSF	1.4914	1.1400	1.6722	0.9262	1.1872
HuberRTSF (δ=0.5)	1.1625	0.7242	1.4464	0.8543	0.5898
HuberRTSF (δ=1)	1.1922	0.7653	1.6250	0.9064	0.7701
HuberRTSF (δ=1.5)	1.2193	0.7988	1.6740	0.8972	0.8586
HuberRTSF (δ=2)	1.2362	0.8386	1.6689	0.8993	0.9078
MCRTSF ($\sigma_{1}=3$)	1.2160	0.7901	1.6713	0.8976	0.7932
MCRTSF ($\sigma_{1}=4$)	1.2309	0.8374	1.6668	0.8994	0.8921
MCRTSF ($\sigma_{1}=5$)	1.2518	0.8840	1.6636	0.9024	0.9603
MCRTSF ($\sigma_{1}=10$)	1.3747	1.0322	1.6665	0.9159	1.1077
MCRTSF ($\sigma_{1}=20$)	1.4557	1.1080	1.6703	0.9232	1.1651
MEERTSF ($\sigma_{1}=0.6$)	1.0686	0.7039	1.7351	0.9892	0.7055
MEERTSF ($\sigma_{1}=1.0$)	1.1094	0.6767	1.7255	0.9757	0.4832
MEERTSF ($\sigma_{1}=1.5$)	1.1101	0.6973	1.7215	0.9814	0.5735
MEERTSF ($\sigma_{1}=2.0$)	1.1205	0.7432	1.7186	0.9875	0.6988
MEERTSF ($\sigma_{1}=3.0$)	1.1418	0.8117	1.7192	1.0018	0.8841
MEERTSF ($\sigma_{1}=10$)	1.3407	1.0881	1.7386	1.0654	1.2053

**Table 2:** RMSE comparison under different noise conditions.

Noises: Gaussian noise					
Algorithms	RMSE of $x_{1}$	RMSE of $x_{2}$	RMSE of $x_{3}$	RMSE of $x_{4}$	RMSE of d
RTSF	0.1052 ± 0.0064	0.0581 ± 0.0020	0.1084 ± 0.0065	0.0591 ± 0.0021	0.0068 ± 0.0002
HuberRTSF	0.1052 ± 0.0058	0.0591 ± 0.0019	0.1084 ± 0.0058	0.0601 ± 0.0020	0.0068 ± 0.0002
MCRTSF	0.1051 ± 0.0063	0.0581 ± 0.0020	0.1083 ± 0.0065	0.0591 ± 0.0021	0.0068 ± 0.0002
MEERTSF	0.1134 ± 0.0063	0.0583 ± 0.0021	0.1207 ± 0.0067	0.0601 ± 0.0021	0.0073 ± 0.0002
Noises: Gaussian noise with outliers					
Algorithms	RMSE of $x_{1}$	RMSE of $x_{2}$	RMSE of $x_{3}$	RMSE of $x_{4}$	RMSE of d
RTSF	0.3596 ± 0.0509	0.2981 ± 0.0305	0.3746 ± 0.0518	0.3037 ± 0.0297	0.0407 ± 0.0044
HuberRTSF	0.1827 ± 0.0551	0.1060 ± 0.0224	0.1804 ± 0.0571	0.1105 ± 0.0212	0.0383 ± 0.0043
MCRTSF	0.2768 ± 0.0496	0.1756 ± 0.0249	0.2761 ± 0.0507	0.1758 ± 0.0249	0.0393 ± 0.0041
MEERTSF	0.3375 ± 0.0510	0.1754 ± 0.0312	0.3540 ± 0.0694	0.1814 ± 0.0385	0.0413 ± 0.0042
Noises: Gaussian mixture noise					
Algorithms	RMSE of $x_{1}$	RMSE of $x_{2}$	RMSE of $x_{3}$	RMSE of $x_{4}$	RMSE of d
RTSF	1.4914 ± 0.2411	1.1400 ± 0.1414	1.6722 ± 0.0974	0.9262 ± 0.0598	1.1872 ± 0.1122
HuberRTSF	1.1625 ± 0.0609	0.7242 ± 0.0825	1.4464 ± 0.0812	0.8543 ± 0.0404	0.5898 ± 0.0863
MCRTSF	1.2160 ± 0.0993	0.7901 ± 0.0988	1.6713 ± 0.0333	0.8976 ± 0.0390	0.7932 ± 0.0786
MEERTSF	1.1094 ± 0.0648	0.6767 ± 0.0991	1.7255 ± 0.0338	0.9757 ± 0.0236	0.4832 ± 0.1216
Noises: Gaussian mixture noise with outliers					
Algorithms	RMSE of $x_{1}$	RMSE of $x_{2}$	RMSE of $x_{3}$	RMSE of $x_{4}$	RMSE of d
RTSF	1.5318 ± 0.1298	1.1067 ± 0.1250	1.6939 ± 0.0388	0.9147 ± 0.0412	1.1963 ± 0.1142
HuberRTSF	1.1663 ± 0.0574	0.7315 ± 0.0690	1.4235 ± 0.0438	0.8595 ± 0.0412	0.6092 ± 0.0881
MCRTSF	1.2583 ± 0.0671	0.7943 ± 0.0757	1.6786 ± 0.0285	0.8999 ± 0.0369	0.8052 ± 0.0699
MEERTSF	1.1169 ± 0.0360	0.6955 ± 0.0696	1.7191 ± 0.0173	0.9800 ± 0.0228	0.5067 ± 0.1592

## Data Availability

The simulation data used in this study are available from the corresponding author upon reasonable request.
